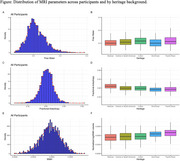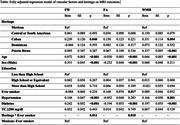# Association Between Vascular Risk Factors and White Matter Integrity Among Diverse Middle‐Aged and Older Hispanic/Latino Adults from the SOL‐INCA‐MRI study

**DOI:** 10.1002/alz70861_108260

**Published:** 2025-12-23

**Authors:** Elmira Agah, Daniela Sotres‐Alvarez, Martha L Daviglus, Fernando Daniel Testai, Haibo Zhou, Carmen R Isasi, Linda C Gallo, Gregory A Talavera, Christian Agudelo, Wassim Tarraf, Hector M Gonzalez, Charles DeCarli, Pauline Maillard

**Affiliations:** ^1^ University of California, Davis, Davis, CA USA; ^2^ University of North Carolina, Chapel Hill, NC USA; ^3^ University of Illinois at Chicago, Chicago, IL USA; ^4^ University of Illinois at Chicago, College of Medicine, Chicago, IL USA; ^5^ University of North Carolina, Chapel Hill, Chapel Hill, NC USA; ^6^ Albert Einstein College of Medicine, Bronx, NY USA; ^7^ San Diego State University, San Diego, CA USA; ^8^ University of Miami Miller School of Medicine, Miami, FL USA; ^9^ Wayne State University, Detroit, MI USA; ^10^ University of California, San Diego, La Jolla, CA USA; ^11^ University of California, Davis, Sacramento, CA USA

## Abstract

**Background:**

Cerebral small vessel disease (SVD) is characterized by white matter hyperintensities (WMH) and microstructural damage detectable via diffusion‐weighted imaging (DWI) parameters such as free water (FW) and fractional anisotropy (FA). Vascular risk factors contribute to white matter damage and cognitive decline. Despite the disproportionate burden of vascular risk factors among Hispanic/Latino individuals, their impact on white matter integrity remains underexplored in this population. We investigated the association between vascular risk factors and MRI measures of white matter changes (FW, FA, and WMH) in cognitively normal Hispanic/Latino adults.

**Method:**

This study was conducted on 1,619 cognitively normal participants from the SOL‐INCA MRI sub‐study cohort of self‐identified Hispanic/Latino individuals, with complete MRI data. MRI data were acquired using standardized 3T protocols, and white matter integrity measures, including FW, FA, and WMH burden, were obtained. Linear regression models were conducted to assess the associations of vascular risk factors (hypertension, diabetes, total cholesterol ≥200 mg/dL, and ever‐smoking) and Hispanic/Latino heritage with white matter integrity measures, adjusting for age, sex, education, and field center.

**Result:**

Analyses showed significant heritage‐based differences in FW and WMH, with Cuban and Puerto Rican participants having higher levels of FW and WMH as compared to Mexican participants (p <0.05). Hypertension and diabetes were associated with increased FW and WMH (p <0.001), and diabetes was associated with lower FA values (p <0.001). No significant interactions were observed between vascular risk factors and heritage except between smoking status and heritage for FW (p = 0.011) and FA (p = 0.010), where smoking status was associated with increased FW and decreased FA among Cuban and Puerto Rican and decreased FA values in Central or South American as compared with Mexican participants (p <0.05).

**Conclusion:**

Our findings indicate heritage‐specific differences in white matter integrity not explained by differences in vascular risk factors. We hypothesize that other factors, such as genetic, cultural, and environmental influences, explain these differences.